# Molecular Detection and Genetic Characterization of Zoonotic Hookworm in Semi-Domesticated Cats Residing in Monasteries in Bangkok, Thailand

**DOI:** 10.3390/tropicalmed8020122

**Published:** 2023-02-15

**Authors:** Pornkamol Phoosangwalthong, Ketsarin Kamyingkird, Chanya Kengradomkij, Wissanuwat Chimnoi, Peter Odermatt, Tawin Inpankaew

**Affiliations:** 1 Center for Agricultural Biotechnology, Kasetsart University, Kamphaeng Saen Campus, Nakhon Pathom 73140, Thailand; 2 Center of Excellence on Agricultural Biotechnology: (AG-BIO/MHESI), Bangkok 10900, Thailand; 3 Department of Parasitology, Faculty of Veterinary Medicine, Kasetsart University, Bangkok 10900, Thailand; 4 Department of Epidemiology and Public Health, Swiss Tropical and Public Health Institute, 4123 Allschwil, Switzerland; 5 University of Basel, 4001 Basel, Switzerland

**Keywords:** hookworms, semi-domesticated cats, PCR, ITS, Thailand, monasteries

## Abstract

Hookworms are the most common parasitic nematodes in the genus of *Ancylostoma* that infect both humans and animals in subtropical and tropical South East Asia. The common hookworm species in cats is *Ancylostoma ceylanicum* which is emerging in Thailand. However, the genetic characterization of hookworms in cats is outdated and insufficiently studied in Thailand. We aimed to investigate the prevalence, risk factors and genetic characterization of hookworm infection in semi-domesticated temple cats in Bangkok, Thailand. A total of 500 temple cat fecal samples were collected from 43 monasteries in 24 districts of Bangkok, Thailand. Polymerase Chain Reaction (PCR) was performed by amplifying the internal transcribed spacer (ITS) gene and mitochondrial cytochrome oxidase c subunit I (*cox 1*) gene. The infection prevalence of hookworm in temple cats was 13.2% (66/500). The highest prevalence was 34.6% in the Bang Khun Thian district, which is located in a suburban area. The risk factor analysis revealed that cats older than one year (OR 2.4, 95% CI 1.1–5.5, *p* < 0.05), lack of veterinary attention (OR 2.9, 95% CI 1.7–4.9, *p* < 0.001) and Bangkok zone (suburban vs. inner city; OR 2.9, 95% CI 1.6–5.4, *p* < 0.001) were significantly increasing hookworm infection risk. All hookworm positive samples were identified as *A. ceylanicum* by ITS gene. Moreover, genetic characterization of *cox 1* gene in *A. ceylanicum* isolates indicated a mix of isolates from humans, cats and dogs. The findings show that temple cats can act as a potential source of zoonotic hookworm parasites for the human and animal population in Bangkok, Thailand. Therefore, appropriate control measures for hookworms in semi-domesticated temple cats as well as prevention measures for hookworms in pet cats and humans should be promoted.

## 1. Introduction

Hookworms are nematode gastrointestinal parasites of mammalian hosts including humans, dogs, and cats distributed worldwide. Hookworm, the genus of *Ancylostoma*, also affected young animals [1], they are generally host-specific species but some species are potentially zoonotic. For the zoonotic potential, the migration of nematode larvae in humans is known as ‘creeping eruptions’ that can lead to an allergic reaction [2] cause by *Ancylostoma braziliense* and *A. caninum*. Whereas, *A. tubaeformae* and *A. ceylanicum* partially develop in the human intestine and cause eosinophilic enteritis. Furthermore, *A. ceylanicum* causes anemia, can be co-infected with fungus and can be fatal in dogs [3]. Besides, *A. ceylanicum* has been reported from stray animals in Asia including dogs and cats in Malaysia [4], dogs in Lao People’s Democratic Republic [5], dogs in India [6], dogs in Cambodia [7], dog in Vietnam [8] and dogs in China [9] with prevalence rates between 43.8 and 94.4%.

Detection of hookworm infection can be performed based on parasitological and molecular methods using Polymerase chain reaction (PCR) [10]. Molecular diagnosis is a useful tool for the identification of hookworm infection in fecal samples. Several gene targets have previously been identified for hookworm identification including ITS, *cox 1*, and *β-tubulin* [11,12,13]. The ITS region is a highly sensitive gene target for the detection and characterization of hookworm at the species level, but it cannot provide adequate genetic resolution on a sub-level [6,12,14,15]. In addition, mitochondrial genes exhibit a more specific sequential variation due to heredity and relatively high evolutionary rates [16], making them suitable for genetic studies of populations such as cytochrome c oxidase subunit 1 encoding gene (*cox 1*). The *cox 1* gene is used for identification purposes, enabling the assessment of the genetic diversity and the genetic structure of hookworm [17,18,19,20].

A considerable number of abandoned cats and dogs in monasteries, public areas, and roadside are present in Thailand. This problem has been unsolved for a long time. Hence, those abandoned cats can be the reservoir of various pathogens including hookworms due to the lack of good sanitation, vaccination, deworming, and regular health care [21]. This active carrier may spread the infective eggs and larvae into the environment. Acquiring zoonotic hookworm infections can occur via direct and indirect transmission. The prevalence of hookworm infection in animals in Thailand was previously reported, but they were mainly conducted in dogs using the direct fecal examination. However, molecular detection and genetic characterization of hookworms in cats have infrequently been studied in Thailand [22,23]. This study aimed to determine the prevalence, risk factors and genetic characterization of hookworm infection in semi-domesticated cats in Bangkok monasteries, Thailand.

## 2. Materials and Methods

### 2.1. Study Areas, Samples, and Data Collection

This study is focusing on the semi-domesticated cats residing in monasteries, in Bangkok metropolitan, the capital of Thailand. Stratified randomization was conducted by selecting 43 monasteries and 24 districts in three zones for the sampling areas. Population size was calculated according to the Taro Yamane method [24] and 500 samples were collected from this study. Two to three monasteries were randomly selected in each district, and five to ten cats were sampled in each monastery. Animal health was examined by a qualified veterinarians prior to sample collection. The form of consent was informed to the animal’s caretakers before sample and data collection. Fecal samples were collected from the rectum, it was immediately placed in a dry bag and stored at 4 °C before transportation to the Department of parasitology, Faculty of Veterinary Medicine Kasetsart University, Bangkok before DNA extraction. A questionnaire was designed to collect animal information on age, sex, free-roaming, veterinary attention, previously deworming and Bangkok zone by interviewing the monks or animal caretakers during sample collection.

### 2.2. DNA Extraction

The previously preserved fecal samples were used for DNA extraction using an E.Z.N.A^®^ Stool DNA kit (Omega Bio-tek Inc., Norcross, GA, USA) according to the manufacturer’s instructions. DNA concentration was measured at 260/230 nm using a BioSpectrometer (Eppendorf AG, Hamburg, Germany) and kept at −20 °C until use in molecular detection.

### 2.3. Molecular Detection of Ancylostoma spp.

#### 2.3.1. Amplification of the ITS Gene

Total DNA samples were screened for the presence of *Ancylostoma* spp. infection with a single PCR to amplify a fragment of 679–690 bp of ITS region using forward primer RTGHF1 and reverse primer RTGHR1 described by [6] (Table 1). Twenty-five microliters of PCR mixture containing 16 µL of ddH_2_O, 0.5 unit of Taq DNA polymerase 0.5 µL (Taq DNA Polymerase, Applied Biological Materials (ABM^®^) Inc., Richmond, BC, Canada), 10 pmol of each forward/reverse primer 1 µL, 2 µL of template genomic DNA, 10×PCR buffer 2.5 µL, 25 mM MgSO_4_ 1.5 µL and 10 mM dNTPs 0.5 µL. The thermocycler conditions included initial denaturation at 94 °C for 2 min, followed by 50 cycles at 64 °C for 1 min, denaturation at 94 °C for 30 s, annealing at 64 °C for 30 s, extension on 72 °C for 30 s and final extension at 72 °C for 7 min. The amplification was performed using a thermocycler (Mastercycler^®^ nexus gradient, Eppendorf AG, Hamburg, Germany). PCR products were identified by electrophoresis with 1.5% agarose gel at 100 V for 40 min.

#### 2.3.2. Amplification of the *cox 1* Gene

*Ancylostoma ceylanicum* hookworm-positive samples were further characterized to a haplotype level by the mitochondrial gene (*cox 1*); a forward primer (Aceycox1F) and a reverse primer (Aceycox1R) to amplify a region of 377 bp as previously described [7] (Table 1). Briefly, 25 µL of the PCR reactions containing 16 µL of ddH_2_O, 0.5 unit of Taq DNA polymerase 0.5 µL (Taq DNA Polymerase, Applied Biological Materials (ABM^®^) Inc., Canada), 10 pmol of each forward/reverse primer 1 µL, 10xPCR buffer 2.5 µL, 25 mM MgSO_4_ 1.5 µL, 10 mM dNTPs 0.5 µL and 2 µL of DNA template. The amplification was initial denaturation at 95 °C for 5 min, followed by 50 cycles of denaturation at 94 °C for 30 s, annealing at 58 °C for 30 s, extension 72 °C for 30 s and a final extension at 72 °C for 7 min. The amplification conditions were controlled by a thermocycler (Mastercycler^®^ nexus gradient, Eppendorf AG, Germany). PCR products were identified by electrophoresis with 1.5% agarose gel at 100 V for 40 min.

### 2.4. Sequencing and Phylogenetic Analysis

All 49 with *cox 1* gene PCR products positive samples were purified using a DNA purification kit (Gel and PCR Purification System, BioFACT^TM^, Daejeon, Republic of Korea) and submitted to the AITbiotech (AITbiotechPte Ltd., Singapore) for sequencing. Check the quality of the sequence using Finch TV (version 1.4.0) and compared using the Basic Local Alignment Search Tool (BLAST). Sequences were manually aligned and consensus sequences were created using BioEdit version 7.2.5 with known sequences from GenBank. A phylogenetic tree was generated with the maximum likelihood (ML) method using the Mega 6 software (www.megasoftware.net (accessed on 21 December 2022)). Bootstrap analyses were conducted using 1000 replicates. References sequences from GenBank including *A. ceylanicum* sequences (accession no. KC247745, KF896596, KF896600, KF896601, KF896605, KP072071, KP072074), *A. caninum* sequence (accession no. NC012309), *A. tubaeforme* sequence (accession no. FR846511) and *A. duodenale* sequence (accession no. NC003415) were constructed and *Giardia lamblia* sequence (accession no. M92053) was used as the outer-group.

### 2.5. Statistical Analysis

All statistical analysis was performed using the R program version 4.05 [25]. The association between exposure variables (sex [male or female], age [<1 year and >1 year], free-roaming [yes or no], veterinary attention [yes or no], previously deworm within 6 months [yes or no] and Bangkok zone [inner city, urban fringe and suburban]) were tested using the chi-square test or Fisher’s exact test. The prevalence of hookworm infections in semi-domesticated cats was summarized using cross-tabulations; Odds ratio (OR) and 95% confidence interval (95% CI) were calculated. The variables in the statistical likelihood ratio at *p* < 0.05 were considered statistically significant.

## 3. Results

### 3.1. Characteristics of the Cat Population

A total of 500 semi-domesticated temple cats were included. Semi-domesticated cats were residing in 43 monasteries, 24 districts, Bangkok, Thailand. There were 42.6% (213) males and 57.4% (287) females. The age group was categorized into two groups including less than one year and more than one year (Table 2). About 50% (268) of cats were free-roaming cats. There were 72.2% (361) and 24.6% (123) cats who received veterinary attention and had previous deworming, respectively. However, all animals in our study were asymptomatic during sample collection. The geographical characteristic of the study areas was categorized as inner city zone (43.6%), urban fringe zone (21.0%), and suburban zone (35.4%) (Table 2).

### 3.2. Prevalence and Distribution of Ancylostoma spp. Infection in Cats

**Figure 1:**
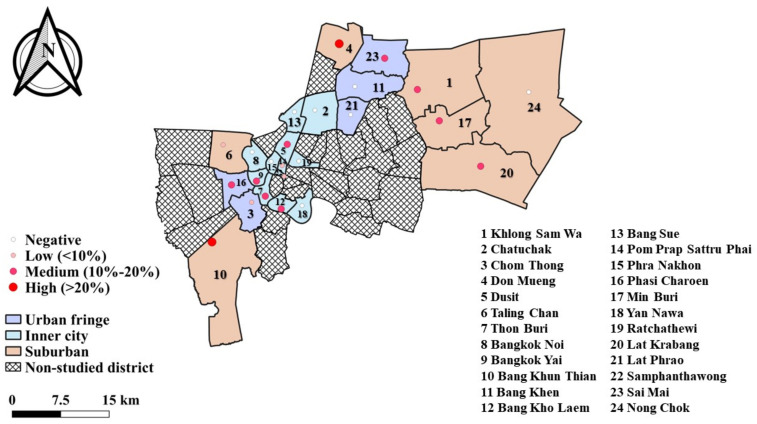
The map of Bangkok showed the geographical characteristic of the study areas into three zones and distribution of hookworm infections in semi-domesticated cats in Bangkok, Thailand.

Amplification of ITS gene revealed that, the overall prevalence of *Ancylostoma* spp. infected in semi-domesticated cats was 13.2% (66/500). The distribution of hookworm infection in semi-domesticated cats was found (53.5%) 23/43 in monasteries were 53.5% (23/43). The highest prevalence of 34.6% was obtained in Bang Khun Thian district, followed by Don Mueang and Khlong Sam Wa, where prevalences were 33.3% and 24.1%, respectively. Hookworm infection in monasteries cats was detected in 16 out of 24 districts (66.7%, Figure 1).

### 3.3. Genetic Characterization of Ancylostoma spp. Infection in Cats

**Figure 2:**
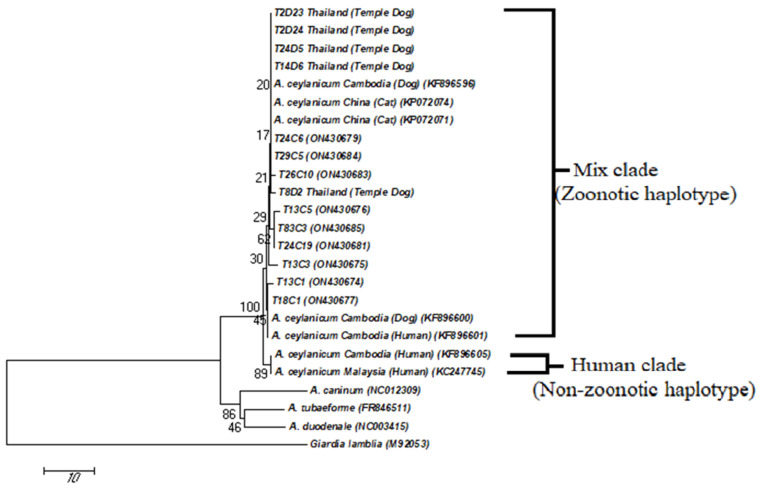
Phylogenetic tree of *A. ceylanicum* based on the nucleotide sequences of a 377-bp fragment of *cox 1* gene by the maximum likelihood (ML) methods (Tamura-Nei parameter model). Number at each branch represents percentage occurrence of clades based on 1000 bootstrap replications of data, with *Giardia lamblia* provided as outgroup.

Of 66 *A. ceylanicum* hookworm-positive samples, 49 (74.2%), were successfully amplified at the *cox 1* gene. All samples were identified as *A. ceylanicum* with 99.9% similarity to the sequence in the GenBank (accession no. KF896604). Phylogenetic analysis showed that all positive *A. ceylanicum* were placed into mix clades together with hookworms from Thai temple dogs in Thailand, Cambodia (dog, cat and human) and China (cats) as shown in Figure 2. Additionally, 12 *A. ceylanicum cox 1* gene sequences received from this study were submitted to the GenBank with the accession no. (ON430677, ON430684, ON430678, ON430674, ON430679, ON430685, ON430675, ON430680, ON430676, ON430681, ON430682, ON430683).

### 3.4. Risk Factors Associated with Hookworm Infection in Cats

Risk factors analysis for hookworm infection in cats demonstrated an increased risk of hookworm infections in adult cats (more than one year) compare to young cats with significant difference (OR 2.4, 95% CI 1.1–5.5, *p* = 0.029). Besides, lack of veterinary attention was significantly difference with hookworm infection in temple cats (OR 2.9, 95% CI 1.7–4.9, *p* < 0.001). While, cats living in suburban are at higher risk to get hookworm infection compare to cats living in monasteries located in urban fringe area with significant difference (OR 2.9, 95% CI 1.6–5.4, *p* = 0.0005) presented in (Table 2).

## 4. Discussion

In Thailand, the prevalence of hookworm infections ranged from 33.2% to 95.8% in cats and dogs, respectively by molecular techniques [22,26,27,28,29]. On the other hand, high prevalence of *A. ceylanicum* in dogs were reported in the monasteries community in Bangkok [27] and Prachinburi province [26]. While in Thai cats, *A. ceylanicum* and *A. caninum* were reported with a prevalence of 23.0% and 92.0%, respectively [26]. In our study, the prevalence of hookworm infection in cats was lower than in the previous studies with no positive for *A. caninum*.

Previous phylogenetic studies have found that several strains of *A. ceylanicum* isolated from humans and dogs clustered in the same group are called zoonotic haplotypes [7,8,12,30]. Therefore, the genetic characterization of the *cox 1* gene has confirmed that the species of hookworm in Thai cats were *A. ceylanicum* and belonged to zoonotic haplotype group, consisting of human-animal mixtures (mix clade) including dog, cat and human in Malaysia, dog, cat and human in Cambodia, temple dogs in Thailand and cats in China as similar to previously studies [7,29]. This finding demonstrated that temple dogs and cats share the same genotype of *A. ceylanicum*. Therefore, the chances of getting hookworms are high among children, nun, monks and people living nearby due to walking barefoot in Thai temples. Hookworm eggs and infective larvae can contaminated soil and be able to transmit to humans and animals through direct contact and walking barefoot in the endemic areas [31].

Risk factor analysis revealed that adult cats were more likely to be infected with hookworms. As indicated by the previous report [32], the age group could be a factor influencing the risk of exposure to hookworm infection and transmission of ancylostomiasis through direct and indirect transmission. This observation could be attributed to the fact that adult cats can go out freely for food or mating. They not only defecate anywhere, but also obtain eggs and larvae from the contaminated environment especially when they live in the same setting as dogs. Hence, previous report found a higher infection rate of hookworm eggs in the temple ground, probably related to the fact that temple ground being exposed to stray animals defecating in the temple areas [33].

In addition, hookworm infection was associated with a lack of veterinary attention. The lack of veterinary care such as vaccination and deworming affect the health and welfare of animals and also adversely affects the health of the owner and others. Hence, vaccination and deworm programs should be preform in regular basis to control and prevent cats from infection and contamination of hookworm in the temple area. This study also found that cats living in the suburban are at higher risk of hookworm infection than cats who live in the urban fringe area similar to previous study in temple dogs from Thailand [29]. Further studies in humans living in the same temples with positive cats might be needed to discover the relation between animals and humans with hookworm transmission. Such information can be used to raise public health awareness and knowledge of humans, animals and environments interaction as part of the One Health approach.

## 5. Conclusions

This study demonstrated the genetic characterization is *A. ceylanicum* in cats residing in Bangkok temple Thailand. Although hookworm infection prevalence in cats are relatively low and asymptomatic, eggs can still be shed with feces and contaminate the environment resulting in an important zoonotic infection risk. Likewise, this result provides an update on molecular prevalence of zoonotic hookworm species infection in semi-domesticated temple cats in Bangkok and may be useful for raising awareness in better control and prevention of hookworm in animals in the monasteries as well as the other public areas in Bangkok, Thailand.

## Figures and Tables

**Table 1:** Primers using for single PCR for the characterization of *Ancylostoma* spp. in stool from semi-domesticated cats.

Target Gene	Primer Name	Product Size	Reference
ITS	RTGHF1: 5′-CGTGCTAGTCTTCAGGACTTTG-3′ RTGHR1: 5′-CGTTGTCATACTAGCCACTGC-3′	679–690	[6]
*cox 1*	Aceycox1F: 5′-GCTTTTGGTATTGTA-AGACAG-3′ Aceycox1R: 5′-CTAACAACATAATAAG-TATCATG-3′	377	[7]

**Table 2:** Risk factor association with hookworm infected in semi-domesticated temple cats, Bangkok, Thailand.

Factor	Number of Cats	Hookworm Positive (%)	Chi-Square χ^2^	Odds Ratio (95% CI)	*p*-Value
Sex			0.14		0.708
Male	287	36 (12.5)		1.0	
Female	213	30 (14.1)		1.1 (0.7–1.9)	
Age			4.80		0.029
Less than one year	104	7 (6.7)		1.0	
More than one year	396	59 (14.9)		2.4 (1.1–5.5)	
Free-roaming			1.67		0.196
Yes	268	30 (11.2)		1.0	
No	232	36 (15.5)		1.5 (0.9–2.5)	
Veterinary attention			15.04		0.0001
Yes	361	34 (9.4)		1.0	
No	139	32 (23.0)		2.9 (1.7–4.9)	
Previous deworming			0.72		0.403
Yes	123	13 (10.6)		1.0	
No	377	53 (14.0)		1.4 (0.7–2.6)	
Bangkok Zone			12.00		0.0005
Inner city	218	18 (8.3)		1.0	
Urban fringe	105	11 (10.5)		2.3 (1.1–4.7)	
Suburban	177	37 (20.9)		2.9 (1.6–5.4)	

## Data Availability

The data presented in this study are available upon request from the corresponding author.
